# Deltoid ligament injury: thinking beyond the ankle – an expert opinion

**DOI:** 10.1530/EOR-2025-0268

**Published:** 2026-09-01

**Authors:** Catherine Fleury, Antoine Acker, Victor Dubois-Ferrière, Mathieu Assal

**Affiliations:** ^1^ Centre Assal, Genève, Switzerland; ^2^ Fondation Forte, Genève, Switzerland; ^3^ Faculté de Médecine, Université de Genève, Genève, Switzerland

**Keywords:** deltoid ligament, ankle trauma, ankle fracture, medial ankle instability, multiligament ankle instability, progressive collapsing foot deformity, spring ligament, flatfoot

## Abstract

- Deltoid ligament (DL) injuries are common in ankle trauma, yet their optimal management remains debated.
- Most trauma literature considers DL injury as an isolated ankle problem, whereas foot-and-ankle specialists view the ankle and the foot as a complex. This disconnect leaves a critical knowledge gap that our work aims to address.
- As we know, every post-traumatic collapsing foot deformity involves a DL injury, but not every DL necessarily progresses to such a deformity.
- We introduce the concept of the DL as a tri-articular stabilizer within the medial ankle ligament complex, contributing to the stability of the tibio-talar, subtalar (ST), and talo-navicular (TN) joints.
- Neglecting this broader stabilizing role may lead to irreversible foot sequelae, such as post-traumatic plano-valgus or plano-abductus deformities. Therefore, DL injuries should be systematically recognized in both acute and chronic settings, whether treated conservatively or surgically.
- In this expert opinion, we synthesize current anatomical, biomechanical, and clinical evidence together with our experience to outline key diagnostic principles and therapeutic considerations for the management of DL injuries.

## Introduction

Ankle injuries represent some of the most common joint traumas encountered in clinical practice (1, 2, 3, 4). Yet, despite their prevalence, several fundamental questions remain unresolved – particularly concerning the deltoid ligament (DL). Recent years have witnessed a growing interest in the DL, particularly its role in ankle stability and surgical decision-making (1, 2, 5, 6, 7). However, this resurgence of attention has remained largely ankle-centric, often ignoring the DL’s broader function within the medial ankle–foot complex. From a biomechanical and clinical perspective, the DL does not merely stabilize the talus within the mortise – it also contributes critically to maintaining proper alignment of the foot (8, 9).

This broader implication of the DL, well recognized in the foot and ankle literature (8, 9, 10, 11, 12), has not yet been adequately integrated into the trauma literature, creating a true ‘no man’s land’ between these two domains. As a result, the long-term consequences of the DL on the foot itself – including the medial column and the hindfoot – remain underappreciated. A likely explanation lies in the traditional Johnson and Strom classification, which equated posterior tibial tendon dysfunction with flatfoot deformity, thereby neglecting the broader spectrum of underlying causes – in the present context, DL injury – that contribute to the development of a progressive collapsing foot deformity, a concept only recently formalized in 2020 (13).

The present expert opinion aims to bridge this conceptual gap by reconsidering DL injury through the lens of the ankle–foot continuum. While this work reflects an expert viewpoint, it is firmly grounded in the current body of evidence, drawing on recent anatomical, biomechanical, and clinical studies to emphasize the DL’s tri-articular configuration and its critical role in maintaining both ankle stability and foot alignment (10). Recognizing this integrated function may be key to preventing post-traumatic deformity and improving outcomes.

## Mechanism of injury

Recent evidence suggests that DL injuries in the context of ankle fractures may be far more common than historically appreciated. In a landmark arthroscopic study, Hintermann *et al.* identified deltoid involvement in up to 40% of acute ankle fractures, challenging earlier assumptions that largely dismissed its clinical relevance (11). One of the most universally used classifications in ankle fractures is the Lauge-Hansen system (14). This radiographic classification is based on the position of the foot at the moment of impact and the direction of the deforming force. Following its publication in the 1950s (14), numerous researchers attempting to recreate the Lauge–Hansen experiments achieved variable results. On the one hand, Warner and colleagues reported a 94% concordance between Lauge-Hansen predictions and both MRI and intraoperative findings in a series of 300 operatively treated ankle fractures, whereas other studies have demonstrated markedly lower levels of concordance (15, 16). Based on these findings, DL rupture typically occurs during supination–adduction (SAD) stage II and supination–external rotation (SER) stage IV injuries. Conversely, when the foot is pronated, DL rupture tends to appear at the very onset of the mechanism – specifically in pronation–external rotation (PER) stage I and pronation–abduction (PAB) stage II.

Rammelt & Boszczyk (16) emphasized that such fractures should not be regarded merely as isolated bony injuries, but rather as the structural manifestation of an underlying ligamentous failure or as the weakest link in the overall injury mechanism. This perspective places acute ankle sprains within the same pathomechanical continuum as fracture injuries. MRI studies of ankle sprains have demonstrated DL involvement in nearly half of cases, with a significantly increased risk of medial ankle ligament complex (MALC) injury when a complete anterior talofibular ligament (ATFL) rupture is present (17). However, the correlation between MRI findings and clinical relevance in this context remains uncertain. Given the high prevalence of acute lateral ankle sprains, the true incidence of MALC injuries in this context is likely underreported. As in the setting of syndesmotic injuries, as mentioned and demonstrated by Gaube *et al.* (18), instable syndesmotic lesions can occur in association with a concomitant DL injury. However, there appears to be significant heterogeneity regarding the severity of both syndesmotic and DL involvement. Nevertheless, isolated DL injuries are rare, representing only 3–4% of ankle injuries, usually following an eversion mechanism (3, 4).

In the acute trauma setting, ruptures most commonly involve both the superficial and deep layers of the DL (17, 19) when associated with concomitant lesions. However, some authors have suggested that, theoretically, the deep deltoid ligament (dDL) – being shorter – may be the first to fail (20). Most tears occur at the medial malleolar attachment, while avulsions from distal insertions and midsubstance tears are reported less frequently (8). In chronic conditions associated with medial ankle instability, the intermediate portion of the dDL is most often affected. Conversely, in cases of rotational ankle instability, the anterior portion of the DL – comprising the anterior dDL and part of the superficial DL (sDL) – tends to be injured due to excessive internal rotation forces that overload these layers (20).

## Anatomy review

**Figure 1:**
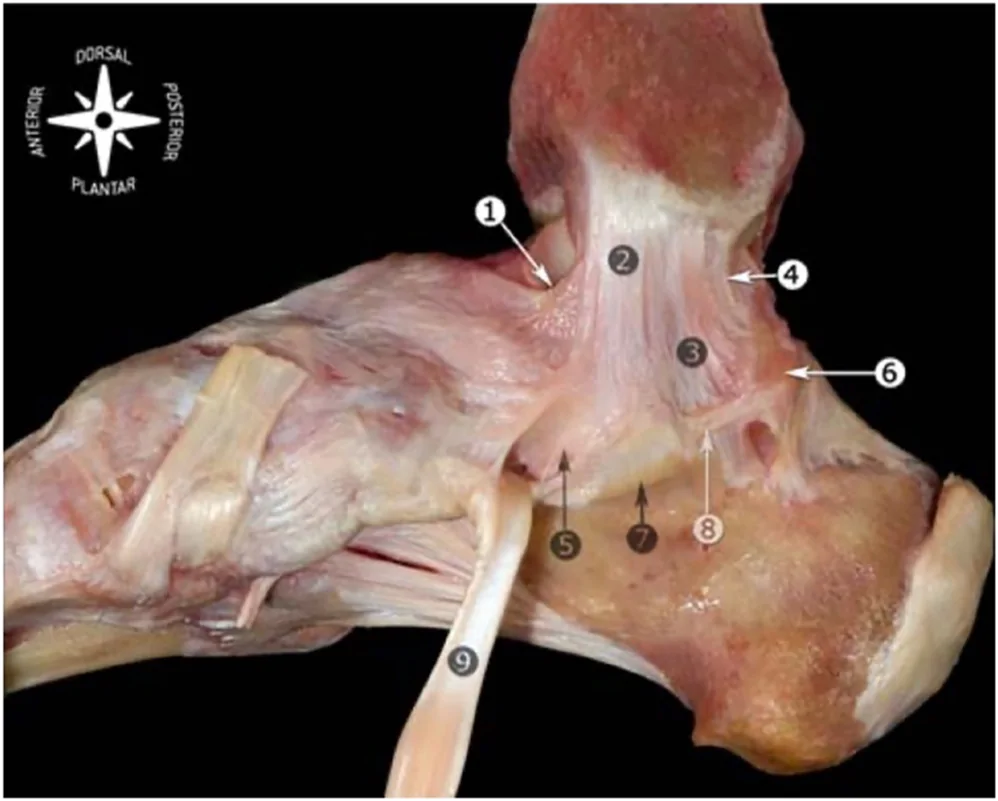
Medial view of the anatomic dissection of the main components of the medial collateral ligament. 1 – Tibionavicular ligament; 2 – tibiospring ligament; 3 – tibiocalcaneal ligament; 4 – deep posterior tibiotalar ligament; 5 – spring ligament complex (superomedial calcaneonavicular ligament); 6 – medial talar process; 7 – sustentaculum tali; 8 – medial talocalcaneal ligament; 9 – tibialis posterior tendon. Copyright: Golanó *et al.* (35)

**Figure 2:**
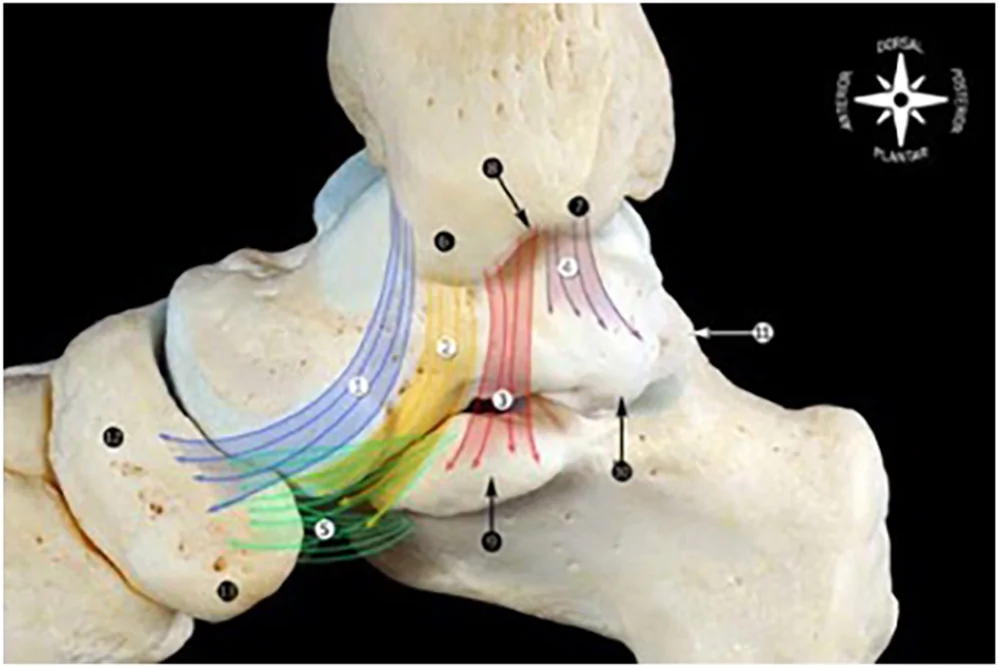
Schematic representation of the main components of the deltoid ligament (DL) crossing three articulations (tri-articular concept): TT, ST, and talo-navicular (TN) joints. The morphology of the medial malleolus is helpful to understand the origins of the medial collateral ligament. In the medial view, two areas or segments (colliculi) can be seen, separated by the intercollicular groove. 1 – Tibionavicular ligament; 2 – tibiospring ligament; 3 – tibiocalcaneal ligament; 4 – deep posterior tibiotalar ligament; 5 – spring ligament complex (plantar and superomedial calcaneonavicular ligaments); 6 – anterior colliculus; 7 – posterior colliculus; 8 – intercollicular groove; 9 – sustentaculum tali; 10 – medial talar process; 11 – lateral talar process; 12 – navicular; 13 – navicular tuberosity. Copyright: Golanó *et al*. (35)

The DL is a complex and robust medial stabilizer of the ankle–foot unit (Fig. 1). Too often regarded solely as an ankle stabilizer, the DL should instead be recognized as a tri-articular medial stabilizer, critically supporting the tibio-talar (TT), sub-talar (ST), and talo-navicular (TN) joints, thereby ensuring both ankle and foot stability (17) (Fig. 2). Extending from the medial malleolus to the talus, calcaneus, and navicular, it is composed of two layers. The dDL, only crossing the TT joint, primarily restrains talar external rotation and prevents lateral translation of the talus, whereas the sDL provides support to the three articulations described above, mainly resisting hindfoot eversion, and supports the medial column of the foot at the TN joint (8, 21, 22). A valgus tilt of the ankle occurs only when both sDL and dDL components of the DL are ruptured (20, 21). Beyond its well-recognized role in countering valgus and external rotation stresses at the ankle, the DL also contributes – though less frequently emphasized – to maintaining hindfoot alignment in both the coronal and axial planes as part of the MALC (10). This functional unit, linking the sDL to the spring ligament through the tibiospring ligament, is fundamental for maintaining foot alignment. The MALC plays a key role in supporting the talar head and stabilizing the peritalar joints. Alongside the posterior tibialis (PT) tendon, which serves as a secondary dynamic stabilizer, the MALC plays a key role in maintaining the integrity of the medial arch (17, 23, 24).

Recognizing the DL as a tri-articular stabilizer explains why its insufficiency can lead not only to ankle instability but more frequently to progressive hindfoot and medial column collapse. This integrated understanding is essential to guide accurate diagnosis and appropriate management.

## The missing outcome

To this day, despite numerous studies, trauma-related publications focus on the ankle stability but rarely explore the downstream consequences of DL injury on foot morphology and alignment (1, 2, 5, 6, 7). In contrast, the foot and ankle fields consistently emphasize the risk of post-traumatic collapsing foot deformity and medial instability following injury to the DL and/or the spring ligament (1, 8, 10). This discrepancy underscores a persistent conceptual gap between the trauma literature and the foot and ankle domain.

Focusing solely on the TT joint neglects the deltoid’s essential role within the ankle–foot complex, where it contributes to the integrity of ST and TN joints. This broader anatomical function raises an important clinical consideration and new perspective for research. Furthermore, although several imaging modalities exist for assessing DL injuries (discussed below), nearly all remain centered on the ankle joint. Very few systematically explore the foot, particularly the TN and ST, despite their direct biomechanical and anatomical dependence on the DL. This paradigm is based on the implicit assumption that apparent ankle stability implies foot stability. Such an assumption may be misleading, as it neglects the distal repercussions of DL injury on the medial column and hindfoot, thereby underestimating the full extent of dysfunction.

## The introduced concept of tri-articular implication of the DL

While the MALC has been extensively described from an anatomical and biomechanical standpoint (17, 23, 24), its integrated function across the ST and TN joints remains largely absent from trauma-related discussions.

Only one study, by Pasapula *et al.* (25), specifically explored this relationship. Analyzing ankle fractures with a complete DL rupture identified by medial clear space (MCS) widening, they found a strong correlation between DL rupture and concomitant spring ligament failure. This finding is pivotal, as it extends the consequences of medial ankle trauma beyond the TT joint to the medial arch of the foot. In their cohort, spring ligament laxity was frequently associated with first-ray instability, predisposing patients to planovalgus deformity.

Although retrospective in nature, this study underscores a critical yet often overlooked dimension: DL injuries may not only destabilize the ankle but also initiate a cascade compromising foot alignment and function. We therefore propose a broader interpretation of the deltoid’s role through its tri-articular configuration (Fig. 2), in which the ligament contributes to maintaining the medial arch at the TN joint – where MALC injury predisposes to post-traumatic plano-abductus deformity – and to preserving hindfoot alignment at the ST joint by preventing post-traumatic plano-valgus deformity. These represent two distinct, clinically relevant conditions. This broader perspective underscores the importance of recognizing DL injury as a multi-joint problem rather than an isolated ankle lesion.

## Current evidence and expert-based strategies for DL injury

### How to make the diagnosis of DL injury in the acute trauma setting?

Schrempf *et al.*, in their systematic review, raised a key question regarding the diagnosis of DL injuries (26). They emphasized the lack of a standardized diagnostic approach, which represents a major gap in the current management of these lesions. In our opinion, the diagnosis should rely on a combination of different tests, as no single test can currently be considered the gold standard. Notably, little attention has been paid to the DL’s role in foot stability, while almost all diagnostic tests described in the literature – and discussed here – focus exclusively on the ankle.

Diagnosis begins with a history of the mechanism and a thorough physical examination: patients with a DL rupture typically present pain along the medial gutter or the course of the DL, sometimes associated with medial malleolar ecchymosis or edema. Although these signs show low sensitivity and specificity, they remain clinically useful (27).

Standard radiographs remain the initial imaging modality, yet their diagnostic value is limited: although mortise and stress views can identify MCS widening or reveal a medial malleolar fleck sign, these findings lack sensitivity and often underestimate the true extent of deltoid insufficiency (20, 28). MRI, despite being the only non-operator-dependent technique capable of directly visualizing soft-tissue structures, has not shown consistent added value in the acute fracture setting (19, 28). It performs poorly in distinguishing partial from complete DL tears, does not reliably assess the severity of MALC injury, and remains insensitive to dynamic instability (26). Ultrasound offers the advantage of dynamic visualization and has demonstrated promising accuracy in selected studies, but its diagnostic reliability is highly operator-dependent, limiting its routine use in acute fracture cases (17). Importantly, all these diagnostic modalities – radiographs, MRI, and ultrasound – are centered on assessing TT instability and do not evaluate the downstream impact of DL injury on ST and TN stability, nor on overall foot alignment.

Diagnosing an acute DL rupture remains a highly nuanced process – one that relies not on a single defining sign, but on a constellation of subtle, interdependent findings. No solitary clinical test, radiographic parameter, or imaging modality has yet demonstrated sufficient accuracy to stand as a gold standard. Instead, the diagnosis emerges from the careful integration of multiple elements: the clinical presentation, targeted physical examination, standard and stress radiographs, dynamic assessment when appropriate, and, in selected cases, intraoperative evaluation and direct surgical visualization. This multifactorial approach reflects both the anatomical complexity of the MALC and the current limitations of available diagnostic tools. Until evidence-based guidelines are established, clinicians must rely on a combination of subtle but meaningful findings, as it is their cumulative weight – rather than any single sign – that supports the diagnosis of DL rupture.

### How to make the diagnosis of DL injury in the chronic trauma setting

**Figure 3:**
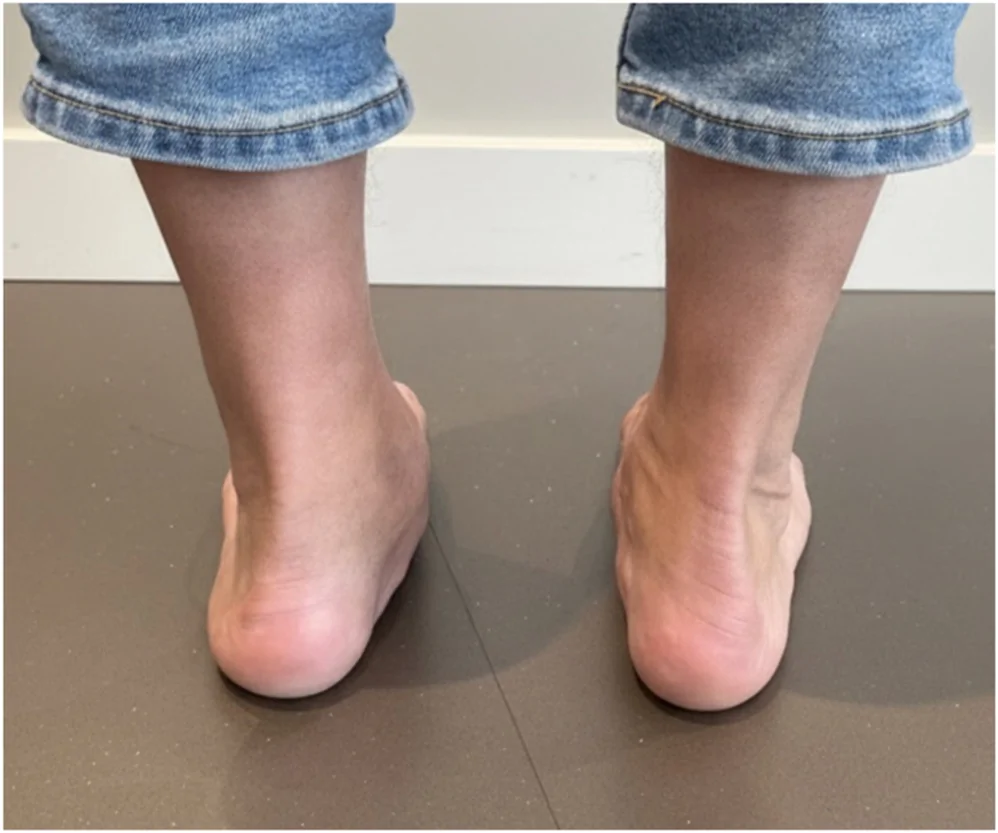
A 37-year-old patient with a bimalleolar-equivalent fracture of the left ankle treated by fibular fixation and rigid syndesmotic stabilization. One year post-injury, he reported persistent medial ankle pain along the deltoid ligament (DL) course and noted a progressive loss of medial arch support. Subsequent investigations confirmed a chronic injury to the medial ankle ligament complex (MALC).

**Figure 4:**
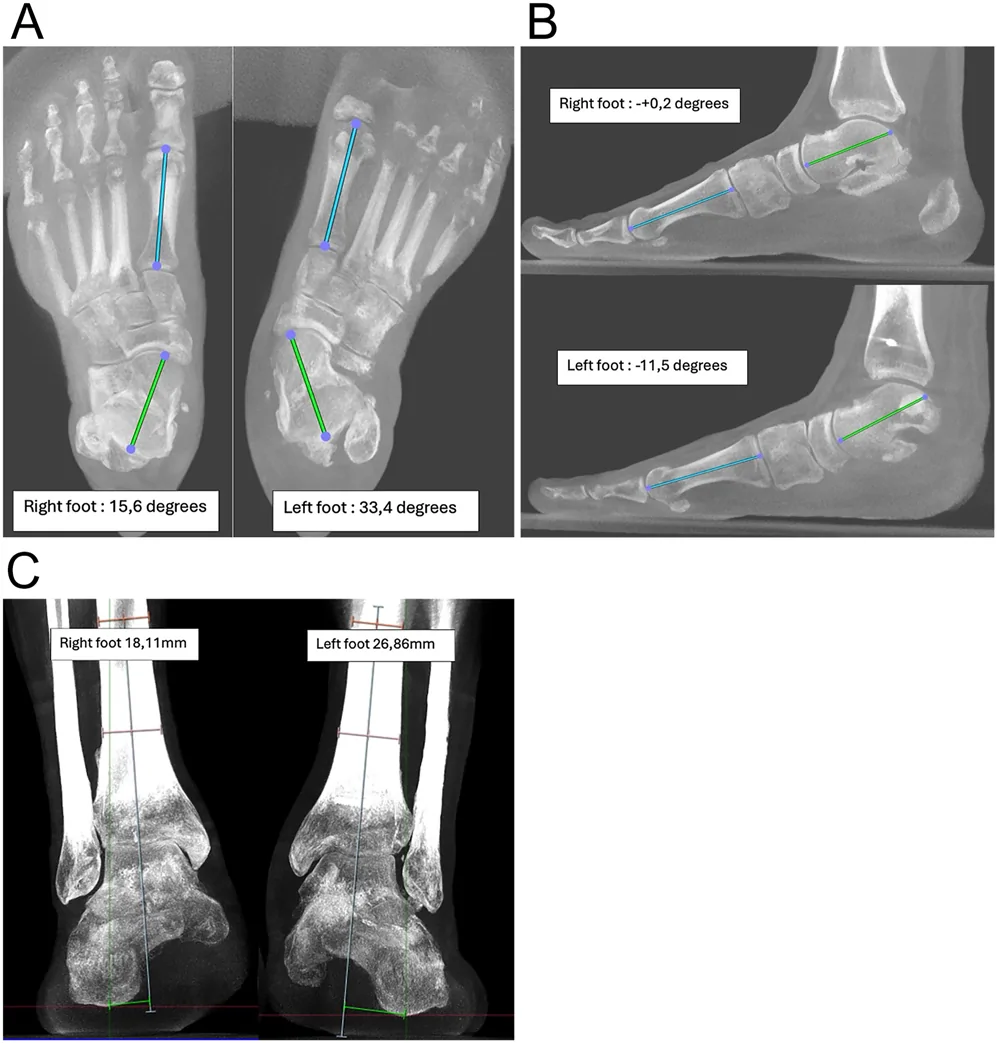
Weight-bearing computed tomography (WBCT) of the same patient shown in Fig. 3. The WBCT reveals segmental deformities marked by forefoot abduction and hindfoot valgus, consistent with a post-traumatic collapsing foot deformity on the left side. (A) Talus–first metatarsal angle – axial view (TFMA-A). (B) Talus–first metatarsal angle (TFMA-S). (C) Hindfoot moment arm (HMA).

In the chronic setting, the key elements remain the patient’s history and clinical examination. As noted by Hintermann *et al.* (23), patients with medial ankle instability often report a pronation deformity of the foot since trauma, recurrent episodes of ‘giving way’, or persistent medial gutter pain (Fig. 3). On physical examination, comparison with the contralateral side may reveal hindfoot valgus or loss of the medial arch with forefoot abduction depending on the extent of DL involvement within what we call its *tri-articular configuration*. In some cases, patients can clearly demonstrate instability through pronation of the foot; if the PT tendon is intact, activation provides dynamic stabilization and corrects the deformity. In our opinion, this remains the most reliable way to establish the diagnosis, as MRI has not yet proven useful. Even though most of our patients in this situation already undergo MRI, we find it difficult to draw firm conclusions regarding the DL and the MALC (29). MRI is less reliable in detecting ligament rupture/insufficiency especially due to the dynamic assessment of the biomechanics (4). Conventional weight-bearing radiographs or weight-bearing computed tomography (WBCT) can show segmental deformities (4) (Fig. 4).

### What about arthroscopy in DL rupture?

Although arthroscopy has gained considerable interest over the past decade, our experience suggests that it provides little additional benefit. Since only a portion of the dDL is intra-articular (24), we remain unconvinced of the added value of this intraoperative step. In a cadaveric study by Guelfi *et al.* (30), arthroscopic exploration of 12 fresh-frozen ankles demonstrated the feasibility of repairing a fascicle of the dDL. Anterior ankle arthroscopy allowed visualization and access to the intermediate portion of the deep layer, which was found to be reachable. Moreover, the fibers observed arthroscopically were found to correlate with the tibiospring fascicle of the sDL. However, in this study, the correlation between these two specific DL fascicles and foot–ankle stability has not been investigated. Moreover, the authors explained that, theoretically, it is the dDL that is injured first, as it is shorter and therefore subjected to higher initial tension. In another study by the same authors (20), they referenced prior work on small cohorts reporting excellent clinical outcomes following arthroscopic repair for rotational ankle instability. One particularly promising result comes from a cohort of seven patients with dynamic medial ankle instability who underwent an all-inside arthroscopic repair of the DL. At the final follow-up, all patients reported subjective improvement in ankle stability, and the sensation of medial arch collapse had resolved. Arthroscopic technique is promising and gaining popularity, particularly due to its less invasive nature. However, stronger clinical evidence is still needed before it can be widely adopted as standard care.

### Should the DL be repaired in the acute trauma setting?

When considering medial ankle sprains, the management algorithm appears better defined, largely thanks to the work of Hintermann and colleagues (9, 23, 31), who have clarified diagnostic criteria and therapeutic strategies. However, in the setting of ankle fractures – particularly the so-called bimalleolar equivalent, significant controversy still persists on the impact of DL rupture. Importantly, these criteria remain centered on the ankle joint. They do not account for the downstream instability that DL rupture may induce at the ST and TN joints, nor its potential impact on foot alignment. This limitation reinforces our systematic approach to DL repair.

From our experience, we systematically repair every DL rupture associated with ankle fractures. The currently accepted criteria in the literature are once again ankle-centric (21). They include failure to reduce the mortise due to DL interposition, abnormal MCS post-fibular plating with external rotation stress test (20, 28), and abnormal valgus tilt greater than 7 degrees (21) as these lesions are inherently unstable. In our opinion, since the patient is already in the operating room, preventing such consequences comes at virtually no additional risk: in our series, no complications have been related to DL repair. A meta-analysis corroborated our experience, showing no statistically significant difference in overall complication rates (7).

In the context of the so-called bimalleolar equivalent, the first step in our algorithm is to fix the fibula, ensuring restoration of its length. In our practice, we find it useful to distinguish this type of ankle fracture into two distinct patterns: either as a trans-fibular fracture associated with DL injury, located distal to the syndesmosis and therefore without syndesmotic involvement, or as a trans-syndesmotic fracture pattern, in which case the syndesmosis is also compromised. In the first scenario, testing is performed, as described above, after fibular plating followed by DL repair and subsequent re-testing to assess the repair. In the second scenario, the same steps are applied, except that they are carried out after both fibular plating and syndesmotic screw fixation.

**Figure 5:**
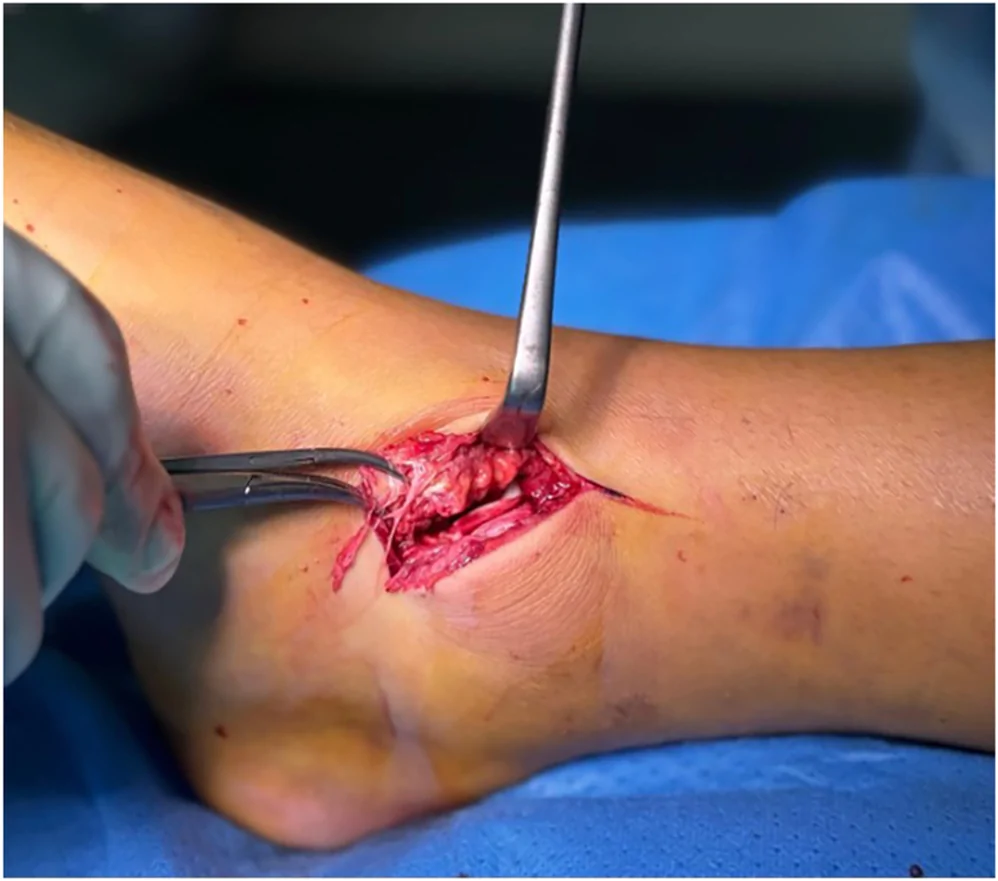
Complete rupture of the deltoid ligament (DL) evidenced by a bare medial malleolus in a 34-year-old patient who sustained a bimalleolar-equivalent fracture of the right ankle.

We perform a longitudinal incision along the anterior aspect of the medial malleolus, which can be extended distally toward the spring ligament and the PT tendon as needed. The anterior incision offers a better visualization of the DL complex, facilitating evaluation of both its sDL and dDL layers during exploration. Upon exposure, the medial malleolus can be found bare (Fig. 5).

In cases of isolated MALC injury, our algorithm is based on the publication of Hintermann (9). We consider that when there is marked swelling and edema around the DL, combined with positive MRI findings confirming DL injury, primary surgical intervention is indicated. Such cases are rare but, in our view, are best managed with primary repair followed by postoperative immobilization in supination for six weeks to protect the repair.

In most cases, however, the lesion corresponds to a partial tear and is treated conservatively (9, 23). We immobilize the ankle in supination with a cast and perform the first follow-up at three weeks to reassess the patient and renew the cast. A further three to five weeks of immobilization is then carried out, in a neutral position with progressive weight bearing. Patients are monitored for six months, and the use of an insole is recommended on a case-by-case basis.

Whether in the setting of a bimalleolar-equivalent fracture or a high-grade DL sprain, we favor primary repair to safeguard the integrity of the foot–ankle complex. In our experience, this approach reliably restores medial stability across the DL’s tri-articular configuration without increasing complication rates.

### Is syndesmotic fixation in lateral malleolus fracture sufficient in the presence of deltoid rupture?

This question is difficult to answer, as the syndesmosis is likely involved to some extent in the injury mechanism, according to the Lauge-Hansen classification described earlier (14, 15). As mentioned above, Gaube *et al.* (18) demonstrated that instable syndesmotic lesions can occur in association with a concomitant DL injury. However, there appears to be significant heterogeneity regarding the severity of both syndesmotic and DL involvement.

In our opinion, when a DL rupture is present, syndesmotic fixation alone will not address the underlying problem related to the DL injury (2). A cadaveric study by Mococain *et al.* (32) evaluated in nine cadaveric legs the effect of rigid syndesmosis fixation alone compared to adding DL repair. The findings of this biomechanical study indicate that DL repair enhances ankle stability in ankle fractures with both syndesmotic and deltoid disruption. This is further supported by a recent systematic review and meta-analysis (33). The authors emphasized that two surgical techniques are most used to address MCS widening in unstable ankle fractures: trans-syndesmotic fixation, which indirectly addresses the MCS, and anatomic DL repair, which directly corrects it. Compared with the trans-syndesmotic fixation group, the deltoid repair group demonstrated significantly lower rates of syndesmotic malreduction (6.5% (4/61) vs 27% (16/59); RR = 0.26, 95% CI: 0.10–0.68) and hardware removal (2.6% (3/115) vs 54.5% (90/165); RR = 0.06, 95% CI: 0.02–0.14). Moreover, syndesmotic fixation restores only tibio-fibular congruency; it does not re-establish medial column stability and peritalar stability, which are directly threatened when the MALC is disrupted.

While the syndesmosis contributes to the overall stability of the ankle mortise, its function remains confined to maintaining the congruency between the tibia and fibula, thereby enhancing ankle stability only. It plays no role in the stabilization of the foot itself. In contrast, the DL extends its stabilizing influence beyond the ankle joint, acting as a key structure for both ankle and foot stability through its connections to the ST and TN joints, as well as its continuity with the spring ligament complex (21, 34).

For these reasons, we believe that syndesmotic fixation cannot be considered a substitute for anatomic DL repair when MCS widening or clinical medial instability is present.

### What is the algorithm for the DL injury in the chronic trauma setting?

Clinically, chronic DL insufficiency presents with persistent medial ankle pain, subjective instability, and, in more advanced cases, signs of progressive hindfoot or medial column collapse. In the chronic setting, it is important to recognize that overuse-related or secondary ankle pathologies are common, as truly isolated DL injuries are uncommon (3, 4). Additional procedures beyond DL repair or reconstruction are often dictated by these concomitant injuries or deformities. The PT tendon may show degeneration, attenuation, or rupture due to the overload induced by MALC insufficiency (11). Similarly, the spring ligament – even if not initially involved – can undergo attenuation as the talar head subluxates (9). In more advanced cases, hindfoot valgus and/or forefoot abduction may develop, representing part of the continuum of post-traumatic foot collapse.

A trial of conservative treatment of 3–6 months is tried, with physiotherapy, particularly with a focus on inverter strengthening, and insoles, braces, or orthoses. Those who fail this regimen undergo surgery.

Isolated repair of the affected ligaments has proven effective in inherently well-aligned feet; however, outcomes differ in cases with long-standing hindfoot valgus and forefoot abduction. In such deformities, ligament repair alone often fails once patients return to full physical or sports activity (9). These situations require, most of the time, additional bony procedures.

In our experience, surgical planning is guided by the type of deformity – whether abductus of midfoot or valgus of hindfoot. For plano-abductus, we perform a vertical plasty of the spring ligament, whereas in planovalgus deformity, the plasty is carried out horizontally and complemented by a plication of the PT tendon. Bony correction to support and protect the repair is considered on a case-by-case basis, either through first metatarsal plantarflexion osteotomy or medializing calcaneal osteotomy, or when the patient already demonstrates a progressive collapsing foot deformity on the contralateral asymptomatic side (9).

Overall, when conservative management fails, surgical planning must account for any associated lesions – including multiligament instability, PT tendon involvement, or other secondary lesions – and the presence and type of foot deformity, whether peritalar subluxation (abductus), hindfoot valgus, or a combination of both (3, 4). These findings determine the need for adjunctive soft-tissue or bony procedures (9).

## Conclusion

The DL must be considered a key component of the ankle–foot complex instead of an isolated ankle stabilizer. Traditional trauma approaches remain largely ankle-centric, overlooking the DL’s essential contribution to ST and TN stability. Yet, a true ‘no man’s land’ persists between the trauma and the foot-and-ankle literature, contributing to diagnostic gaps and suboptimal treatment strategies (1, 2, 5, 6, 7). We know that all post-traumatic collapsing foot deformities involve the DL; however, not all DL injuries necessarily evolve into a post-traumatic collapsing foot deformity (8, 9, 10, 11, 12).

Considering the DL as a tri-articular stabilizer provides a more accurate biomechanical framework. Its insufficiency can initiate a cascade of changes affecting the hindfoot and medial column, predisposing patients to post-traumatic plano-valgus or plano-abductus deformities. These downstream effects may be unrecognized as most diagnostic modalities focus on TT alignment, potentially neglecting the broader implications of MALC failure. Integrating the ankle–foot continuum into clinical evaluation is essential. A systematic assessment of ST and TN joints, and identification of associated spring ligament involvement, can improve diagnostic accuracy and outcomes.

In fracture management, DL repair helps restore medial stability across multiple joints and reduces the risk of long-term foot deformity, complementing fibular and syndesmotic fixation when indicated. Given the reasons outlined above and the consequences of untreated DL insufficiency on foot alignment, we believe that it is important to spell out clearly that repairs should be considered more often. Such repairs can be performed at a low morbidity, and a more proactive indication is justified while awaiting publication of more formal outcome data.

## ICMJE Statement of Interest

The authors declare that there is no conflict of interest that could be perceived as prejudicing the impartiality of the research reported.

## Funding Statement

This research did not receive any specific grant from any funding agency in the public, commercial, or not-for-profit sector.
